# Generative deep learning for the development of a type 1 diabetes simulator

**DOI:** 10.1038/s43856-024-00476-0

**Published:** 2024-03-16

**Authors:** Omer Mujahid, Ivan Contreras, Aleix Beneyto, Josep Vehi

**Affiliations:** 1 Modelling, Identification and Control Engineering Laboratory, Institut d’Informatica i Aplicacions, Universitat de Girona, Girona, 17003 Girona Spain; 2 Centro de Investigación Biomédica en Red de Diabetes y Enfermedades Metabólicas Asociadas (CIBERDEM), Girona, Spain

**Keywords:** Type 1 diabetes, Type 1 diabetes

## Abstract

**Background:**

Type 1 diabetes (T1D) simulators, crucial for advancing diabetes treatments, often fall short of capturing the entire complexity of the glucose-insulin system due to the imprecise approximation of the physiological models. This study introduces a simulation approach employing a conditional deep generative model. The aim is to overcome the limitations of existing T1D simulators by synthesizing virtual patients that more accurately represent the entire glucose-insulin system physiology.

**Methods:**

Our methodology utilizes a sequence-to-sequence generative adversarial network to simulate virtual T1D patients causally. Causality is embedded in the model by introducing shifted input-output pairs during training, with a 90-min shift capturing the impact of input insulin and carbohydrates on blood glucose. To validate our approach, we train and evaluate the model using three distinct datasets, each consisting of 27, 12, and 10 T1D patients, respectively. In addition, we subject the trained model to further validation for closed-loop therapy, employing a state-of-the-art controller.

**Results:**

The generated patients display statistical similarity to real patients when evaluated on the time-in-range results for each of the standard blood glucose ranges in T1D management along with means and variability outcomes. When tested for causality, authentic causal links are identified between the insulin, carbohydrates, and blood glucose levels of the virtual patients. The trained generative model demonstrates behaviours that are closer to reality compared to conventional T1D simulators when subjected to closed-loop insulin therapy using a state-of-the-art controller.

**Conclusions:**

These results highlight our approach’s capability to accurately capture physiological dynamics and establish genuine causal relationships, holding promise for enhancing the development and evaluation of therapies in diabetes.

## Plain language summary

New therapies and treatments for type 1 diabetes (T1D) are often first tested on specialized computer programs called simulators before being tried on actual patients. Traditionally, these simulators rely on mathematical equations to mimic real-life patients, but they sometimes fail to provide reliable results because they do not consider everything that affects individuals with diabetes, such as lifestyle, eating habits, time of day, and weather. In our research, we suggest using computer programs based on artificial intelligence that can directly learn all these factors from real patient data. We tested our programs using information from different groups of patients and found that they were much better at predicting what would happen with a patient’s diabetes. These new programs can understand how insulin, food, and blood sugar levels interact in the body, which makes them valuable for developing therapies for T1D.

## Abstract (web summary)

Mujahid et al. develop a type 1 diabetes patient simulator using a conditional sequence-to-sequence deep generative model. Their approach captures causal relationships between insulin, carbohydrates, and blood glucose levels, producing virtual patients with similar responses to real patients in open and closed-loop insulin therapy scenarios.

## Introduction

The terms modelling and simulation (M&S) go hand in hand. Although defined differently by different researchers depending mostly on the field of study, the majority of these definitions are some variant of the interpretation presented by Kaizer et al.^1^, which states that a model is a “representation of a system, entity, phenomenon, or process”, whereas a simulation is “the imitation of a behaviour of a system, entity, phenomenon or process through the exercise or use of a model”. The purpose of M&S is the emulation and approximation of physical phenomena that cannot be directly observed for the purpose of better understanding. It is the process of explaining how an object of interest behaves in an environment. Biomedical simulation tasks may employ some model(s) of a biological system to emulate the physics underlying biological organs in coordination with certain other mechanisms to form a complete simulation environment.

The majority of biomedical simulators use mathematical physiological or pharmacokinetic models to simulate biological phenomena^2^. Such simulators are important for the development and testing of new treatments and therapeutic strategies for different diseases because they offer an inexpensive alternative to patient and animal testing both in terms of time and money. Moreover, they can quickly help identify parts of the device design that may not be effective and prevent the development of adverse circumstances because they are easy to interpret^3^. Although efficient to some extent in the task of approximating phenomena resulting from biological organs, the physiological models do not capture the entirety of a biological process because of various elements that are simply not possible to model^4^. These elements may include certain unmeasured variables affecting the outcomes of a process or external influencing factors such as a patient’s lifestyle choices, routine habits, and environmental factors^5^. Consequently, an intrinsic error is induced in the approximation of physiological models in the form of nonrandom grey noise that compromises the effectiveness of these models in an unavoidable fashion. Since a model is a combination of various parts or compartments, failure to approximate a real scenarios is often unexplained in terms of error-inducing components^6^. Similarly, in the case of an accurate approximation, the reason for success may not be measured deterministically.

Existing type 1 diabetes (T1D) simulators employ physiological models of the glucose-insulin system for the purpose of synthesizing glycaemic scenarios emulating real-life T1D patients^7–9^. Integration of such physiological models with open-loop or closed-loop control methodologies enables the creation of simulation environments that imitate the glucose-insulin relationship of T1D patients. These simulators have made the development and testing of several treatment methodologies possible for T1D patients^10^. However, previously as discussed, the physiological descriptions of the glucose-insulin system are far from perfect, and the T1D simulators based on these models suffer from the induced inaccuracies of the mathematical descriptions. Having no provision regarding patients’ life choices and habits or other life disturbances such as menstruation, depression, and medication, these simulators often fail in scenarios that require higher understanding of the real-life factors affecting diabetics. Therefore, the exploration of approximation techniques that can fully consider all of the factors affecting the glycaemic trends of a T1D patient is necessary. Such an approximation technique needs to consider external factors such as exercise, illness, menstrual cycles, sleep disorders, depression, and medication. Furthermore, the existing T1D simulators have little support for patient behaviour, such as eating habits, alcohol consumption, and lifestyle choices. These factors have a substantial effect on the glycaemic profile of a person, and including these disturbances in a simulator will ensure the high accuracy of the simulation scenario. It is understood that modelling each one of these disturbances individually is an impossible task; however, the effect of these disturbances can be approximated from generated data. The proposed methodology leverages the concept of modelling from data using generic function approximators.

Generic function approximators have been proven to learn complex nonlinear relationships from data. According to the universal approximation theorem, an artificial neural network (ANN) with a hidden layer is capable of learning almost any function given that it is sufficiently wide^11,12^. This means that a neural network designed to learn the probability distribution of data may learn any complex distribution provided that the network is apt enough and a sufficient amount of data is available. Moreover, with a sufficient amount of data, deep neural networks (DNNs) are capable of surpassing mathematical models to better approximate systems^13,14^. Deep generative models are DNNs that are capable of learning the underlying probability distribution of data and then generating novel samples from the learned distribution. The effectiveness of deep generative models in approximating distributions accurately has been demonstrated by several recent studies in various areas of research, including biomedical applications^15–18^.

With reference to probability theory, deep learning models are often divided into the following categories: generative models, discriminative models, and composite models^19^. Discriminative models learn the conditional probability distribution from data, whereas generative models learn the joint probability distribution from data by learning a probability density function over all the samples present in a dataset. Composite models, on the other hand, are a combination of discriminative models and generative models. Deep generative models may either be generative or composite. Evidently, the data obtained from human glucose-insulin systems depict a complex underlying probability distribution, and deep generative models are one of the most suitable methodologies for learning this type of distribution. The field of deep generative models has evolved considerably over the last few years^20,21^. The performance quality of these models is context-dependent such that none could be deemed superior to the other and vice versa. Some of the most popular deep generative models include variational autoencoders, generative adversarial networks (GANs), normalizing flows, diffusion models, and autoregressive models. In this work, we employ a GAN for the task of conditional BG generation. A GAN is chosen over other types of deep generative models because it is specifically set up to optimize generation tasks and generate high-quality data. Other deep generative models, such as variational autoencoders, flow-based models, and diffusion models, all model the latent variable and are not used here because of the problems related to latent variable approximation^22,23^. Moreover, other deep generative models such as deep autoregressive models do not contain an explicit latent space.

The advent of artificial intelligence (AI) has increased the number of biomedical tools based on AI models/techniques, such as machine learning (ML), deep learning (DL), and reinforcement learning (RL)^24,25^. A similar increasing trend is observed in the literature regarding the use of these techniques in diabetes healthcare. Although, in diabetes healthcare, substantial literary evidence exists of the use of these techniques for various purposes such as diagnosis, therapy optimization, recommendation, and education, they are predominantly being used for the prediction of BG values or adverse glycaemic events^26–28^. Apart from prediction, AI-based methodologies have also been extensively used for therapy optimization in diabetes healthcare^29,30^. However, the majority of these applications use discriminative models that are optimized to learn the conditional boundaries in a dataset. In contrast to conditional models, generative models are continually preferred by researchers for data synthesis because of their ability to generate realistic new samples. Studies have shown that for the task of data generation, generative models outperform discriminative models^31,32^. Moreover, generative deep learning has been applied in various fields where the need for synthetic data is considerable. Although these applications are inclined towards perceptual data generation, such as images and music, an increasing number of studies have been using these models for applications in medicine and healthcare^33–35^.

In diabetes healthcare, deep generative models have mostly been used in comorbidity studies, such as studies on diabetic retinopathy, which is diagnosed using image scans^36–39^, or for conditions that could be assessed visually, such as diabetic foot conditions^40^. Recently, several studies have used deep generative models, including GANs, to generate other types of diabetes-related data, such as BG time series^41,42^, tabular data^43^, and electronic health record data^44^. The use of synthetic data generated through GANs has been approached differently by different studies. While some studies used synthetic data to train ML models directly, others have used it to augment datasets to improve the performance of ML/DL models^45,46^. There have also been some research studies that have used GANs to predict future BG values^47^. However, the principles of probability theory and evidence in the literature oppose the use of deep generative models for time series predictions^48^.

This research proposes a strategy of utilizing deep generative models for the task of simulating T1D patient profiles by learning glycaemic trends in the form of glucose-insulin and glucose-carbohydrate relationships from the data. It takes inspiration from two of our prior works in this line^42,45^. The first work provided evidence for the feasibility of using deep generative models for the synthesis of diabetes-related data^45^, and the second work provides a basis for the conditional generation of BG values^42^. In the first work, a conventional vanilla GAN was employed for the synthesis of BG values for T1D patients to augment individual patient datasets and train a nocturnal hypoglycaemia prediction model. The results showed that data augmentation through the GAN improved the results of the prediction model. The second study employs an instantiation of a pixel-to-pixel (P2P) GAN to demonstrate the proof of concept for the generation of conditioned values. Since the P2P GAN architecture is designed for image data, the study contributed with novel translation mechanisms for translating time series data of BG and insulin into images and vice versa. The models were validated by generating time series of BG values conditioned by insulin doses that mimicked real patients, followed by a comparison of different glycemic metrics between real and virtual cohorts. It is important to mention here that after an extensive literature search, no evidence of a deep generative model-based T1D simulator was found. Although GluGAN by Zhu et al. comes closest to the proposed work in terms of the desired aim, its scope is more relevant to our prior works mentioned above^49^. However, the idea of using deep generative models for modelling in simulation environments has been exploited in other fields, such as astronomy^50^, particle physics^51^, spectral analysis^52^, protein folding^53^, and smoke sequence simulators^54^.

The idea of conditional generation of BG values is leveraged by the simulator proposed in this article. The proposed methodology employs a conditional generative adversarial network (CGAN) for the generation of BG values conditioned on the plasma insulin approximation (PI) and the carbohydrate rate of appearance (RA) of T1D patients. The proposal introduces a tailored CGAN methodology using a sequence-to-sequence (S2S) architecture. This study also contributes to the introduction of shifted input-output pairs in training, which facilitates the integration of causality within the proposed simulator. This enables the generated BG values to depict the input insulin and carbohydrates that impact the glycaemic profile over time. Furthermore, the inherent quality of causality guaranteed the compatibility of the proposed model with a closed-loop controller, enabling validation within the context of closed-loop insulin therapy. Consequently, this paved the path for the creation of the simulation environment outlined in this research article. We have demonstrated through a multitherapeutic validation approach that the generated BG values exhibit a dependency on the input PI and RA values, which is consistent with the glycaemic relationships of real T1D patients.

The primary contributions of this study include: firstly, the development of an artificial intelligence-driven T1D simulator featuring both open-loop and closed-loop insulin therapies; secondly, the causal generation of BG values using GANs; and thirdly, the generation of realistic T1D patient profiles, conditioned on insulin and carbohydrate values. We tried to include as many details as possible about the tools and datasets used in the study, problem formulation, and results based on the benchmarks of robustness for AI in healthcare proposed by Diana Mincu and Subhrajit Roy^55^. The rest of the paper is focused on explaining the methodology to generate causal data using CGANs; the results containing the statistical similarity tests, causality analysis, and distribution comparison; and the discussion about the limitations and possibilities of this work.

## Methods

This section considers the approaches, techniques, and models required to devise the proposed methodology. The section starts by first explaining the proposed GAN model that constitutes the major portion of the proposed methodology and then describes the training of this model for the task of learning causal relationships between BG, insulin, and carbohydrate data of T1D patients. The conditional generation of BG data for the synthesis of virtual T1D patients using the trained models is demonstrated at the end of the section.

### Sequence-to-sequence generative adversarial network

**Fig. 1:**
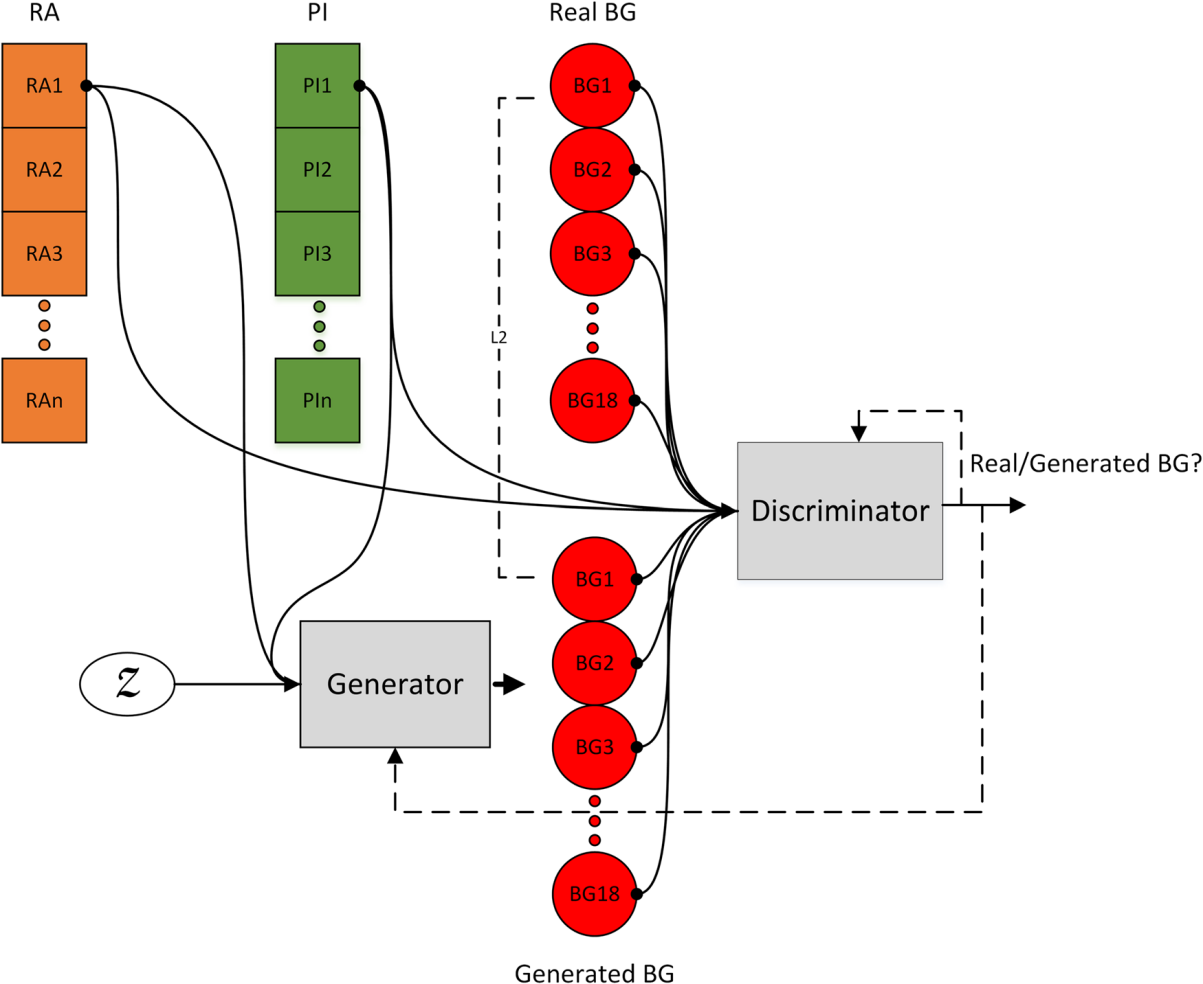
S2S model used for the generation of blood glucose values conditioned on insulin and carbohydrates. The plasma insulin and carbohydrate rate of appearance values are the conditional inputs for the blood glucose values. These conditional inputs are paired up with both the real and generated blood glucose values during training (PI: plasma insulin approximation, RA: carbohydrate rate of appearance, BG: blood glucose, Z: latent space).

This study proposes the use of a CGAN, which is a type of GAN, for the generation of BG samples^56^. A CGAN conditions the generated samples on some other type of input. The conditions are provided as labels to the GAN. This formulation could also be described as a translational model where one form of data (labels) is translated into another form of data (generated samples). When slightly altered, such a model can be transformed into a sequence-to-sequence (S2S) or a pixel-to-pixel (P2P) model^57^. For an S2S model, the CGAN is required to translate an input vector into an output vector. For a P2P model, the CGAN deals with image pixel data. The proposed methodology uses the S2S approach to condition the output array of BG values on the input arrays of insulin and carbohydrates. Figure 1 shows the S2S GAN architecture utilized in the proposed methodology for the generation of T1D patients.

As mentioned above, a GAN is a hybrid deep architecture composed of two DNNs working in a zero-sum game to achieve optimization. These DNNs are the discriminator (*D*) model and the generator (*G*) model. The *D* model in our proposed architecture is presented in Supplementary Fig. 1. As the name suggests, the *D* model discriminates between two sets of samples, such as the real and generated samples. This model is trained on real samples from the dataset and fake samples generated by the generator. Our proposed S2S GAN uses the Wasserstein loss in the *D* model to determine whether a given sample is real. For classification purposes, the real samples are labelled as ‘−1’, whereas the fake samples are labelled as ‘1’. As seen in Supplementary Fig. 1, the *D* model is composed of five 1D convolutional layers of different sizes. 1D convolutional layers are chosen to learn the temporal characteristics of the time-dependent data. The input to the *D* model is a concatenated signal of one sample of PI, one sample of RA, and 18 samples of BG. This combination of signals serves as the input/output configuration required for the S2S GAN. The PI and RA are the input pair, whereas BG is the output. The task of the *D* model is to distinguish between the real and synthetic configurations of these three signals. On the other hand, the *G* model tries to learn the underlying probability of the real dataset based on the feedback it receives from the *D* model and the L2 distance of its output from the ground truth. Supplementary Fig. 2 displays the architecture of the *G* model in our S2S GAN. It takes a three-signal formation of PI, RA, and the latent space (*Z*) samples as input that are passed through a dense layer before being reshaped and concatenated. *Z* is chosen to be a normal distribution. The *G* model is composed of a total of four 1D transpose convolutional layers. For the *G* model, PI and RA are obtained from the real data and condition the transformation of *Z* into BG values. Both *D* and *G* are trained using the Wasserstein loss scheme, which is given in Eq. (1). *W*(*P*_*r*_, *P*_*g*_) is the Wasserstein distance between the real data distribution *P*_*r*_ and the generated data distribution *P*_*g*_. inf denotes the infimum, which is the greatest lower bound. *γ* is a joint probability distribution on the product space of *P*_*r*_ and *P*_*g*_ such that *γ* ∈ Π(*P*_*r*_, *P*_*g*_); that is, *P*_*r*_ and *P*_*g*_ are its marginals. ${{\mathbb{E}}}_{(x,y) \sim \gamma }[\parallel x-y\parallel ]$ represents the expected value of the distance between pairs of points (*x*, *y*) drawn from the joint distribution *γ*. However, calculating the Wasserstein distance in this way is computationally intractable. Therefore, the Kantorovich-Rubinstein duality theorem is used, which transforms the Wasserstein distance (an infimum) into the supremum of a class of Lipschitz functions. This expression is given in Eq. (2).

1 $$W({P}_{r},{P}_{g})=\mathop{\inf }\limits_{\gamma \in \Pi ({P}_{r},{P}_{g})}{{\mathbb{E}}}_{(x,y) \sim \gamma }[\parallel x-y\parallel ]$$

2 $$W({P}_{r},{P}_{\theta })=\mathop{\sup }\limits_{\parallel f{\parallel }_{L}\le K}{{\mathbb{E}}}_{x \sim {P}_{r}}[ \, f(x)]-{{\mathbb{E}}}_{x \sim {P}_{\theta }}[\, f(x)]$$

The GAN objective using the Wasserstein distance is given in Eq. (3). The *G* model tries to minimize it by generating samples that are more likely to be classified as real by the *D* model. The *D* model tries to maximize it by correctly classifying real data samples (*x*) as real and generated samples (*G*(*z*, *y*)) as fake. This encourages the discriminator to be a good critic of the generated samples.

3 $$L({\theta }_{G},{\theta }_{D})=\mathop{\min }\limits_{{\theta }_{G}}\mathop{\max }\limits_{{\theta }_{D}}\left[{{\mathbb{E}}}_{(x,y) \sim {p}_{{{{{{{{\rm{data}}}}}}}}}}[D(x,y)]-{{\mathbb{E}}}_{z \sim {p}_{z}}[D(G(z,y),y)]\right]$$

To ensure that the *D* model obeys the Lipschitz constraint to obtain a maximum gradient, the weights of the models are clipped. The *G* loss is also mixed with an L2 distance to enable *G* to not only fool the *D* but also to be closer to the real output. Equation (4) represents the L2 distance between the generated output *G*(*z*, *y*) and the ground truth *x*.

4 $$L2\,distance:{{\mathbb{E}}}_{(x,y) \sim {p}_{{{{{{{{\rm{data}}}}}}}}},z \sim {p}_{z}}\left[\parallel G(z,y)-x{\parallel }_{2}^{2}\right]$$

The final objective of our S2S GAN is shown by Eq. (5). *λ* is a hyperparameter that controls the importance of the L2 loss term relative to the adversarial loss terms. The losses for both *D* and *G* are averaged over a mini-batch of data. The complete S2S model is trained using paired data with one PI and one RA value mapped to 18 BG values in each pair.

5 $$GAN\,objective:L({\theta }_{G},{\theta }_{D})+\lambda \cdot L2\,distance$$

#### Experimental datasets

We used data from three different cohorts, namely, the Hospital Clínic de Barcelona T1D dataset^58^, the Hypomin dataset^59^, and the Ohio dataset^60^, with 27, 10, and 12 patients, respectively. The Hospital Clínic de Barcelona T1D dataset consists of data from 57.7% female participants and the remaining data are from male participants. The average age, weight, and height of the participants were 46.12 years, 71.28 kg, and 166.80 cm, respectively; the average length of time a patient had diabetes was 31.42 years, and the HbA1C was 7.11. No demographic information could be obtained for the Hypomin dataset; however, the inclusion criteria of the clinical trials consist of patients older than 18 years with T1D on multiple daily insulin (MDI) therapy, a disease duration greater than 5 years, and HbA1C in the range of 6.5–9.5%. In the process of collecting and analysing these datasets, ethical approval and consent were not specifically sought for this study, as they were acquired as part of larger clinical trials conducted by the Hospital Clínic de Barcelona. These trials have already undergone rigorous ethical review processes, and the datasets used in this study were derived from anonymized patient data collected during these clinical trials, where participants provided informed consent for their data to be used for research purposes. Ethical approval for our use of Hospital Clinic de Barcelona T1D dataset and the Hypomin dataset was not required because prior ethical approval allows the use of these datasets as per Spanish law. The Ohio T1DM dataset consists of 41.7% female participants, and the remaining participants were male. The average age was in the range of 36.66–56.66%. The patients in this cohort were on insulin pump therapy. The data used in our implementation contained the BG profiles of each patient along with the insulin and carbohydrate information. Basal and bolus insulin values were first converted into the patient’s PI approximation using Hovorka’s insulin pharmacokinetic model^7^. On the other hand, carbohydrate values that were given in grams in the original dataset were depicted as the RA values using the mixed meal libraries from Ernesto et al.^61^. RA values of meals that fit meal descriptions based on the time of the day the meal were considered, and the number of grams of the meal was chosen to represent a particular meal. In the end, the time series obtained from carbohydrates was a combination of the carbohydrate RAs of all the meals consumed by the patient in a particular time frame.

The inputs and outputs were introduced as pairs to the GAN model. This essentially means that for one input pair, there were a total of 18 values of output BG. The shift was achieved using the ‘roll’ function in Python. The training data were normalized before being subjected to training, which was achieved using MinMaxScaler in scikit-learn. These shifted normalized pair data were then used to train the S2S GAN model.

### Training with recurring data

Our proposed S2S model was trained on PI, RA, and BG values from a T1D patient cohort. To introduce causality between the inputs and the outputs, a shift of 90 min was introduced between the PI/RA pair and BG values. This was done to completely capture the impact of insulin and carbohydrates on the BG profile of a T1D patient. Since the sampling rate of the signals was 5 min, to introduce a shift of 90 min, the shifted pairs were formed by mapping one PI/RA pair value to 18 future BG values. The recurring pairs of data acted as snapshots of the cause-and-effect relationship between them. The entire dataset was first converted into groups of 1 PI/RA pair and 18 BG values. A visualization of the shifted pairs of insulin, carbohydrates, and BG can be seen in Supplementary Fig. 3. The batch size was kept at 1 to introduce more diversity in the samples by enabling the model to learn the minute intricate relationships from each individual pair without generalizing too much over a large batch of data. Moreover, smaller batch sizes are associated with training stability and lower generalization error. This enabled the model to learn the causal relationships among the data trickling through each recurrent sample of data. The S2S GAN model was then trained with the shifted samples for a total of 50 epochs because empirical testing suggested this as the optimal value for all the models to achieve convergence. By following the recommendations of the authors of the Wasserstein GAN (WGAN), the discriminator was trained 5 times more than the generator. Since the batch size was chosen to be 1, it considerably slowed the training process, as the time taken in training for one epoch directly depended on the total number of samples included in the training data. A leave-one-out scheme was used to train the S2S GAN for the entire cohort. In this scheme, a model was trained by removing the data of one patient from the training dataset each time and then used to generate data for the patient not included in the training dataset.

### Synthesis of virtual T1D patients

**Fig. 2:**
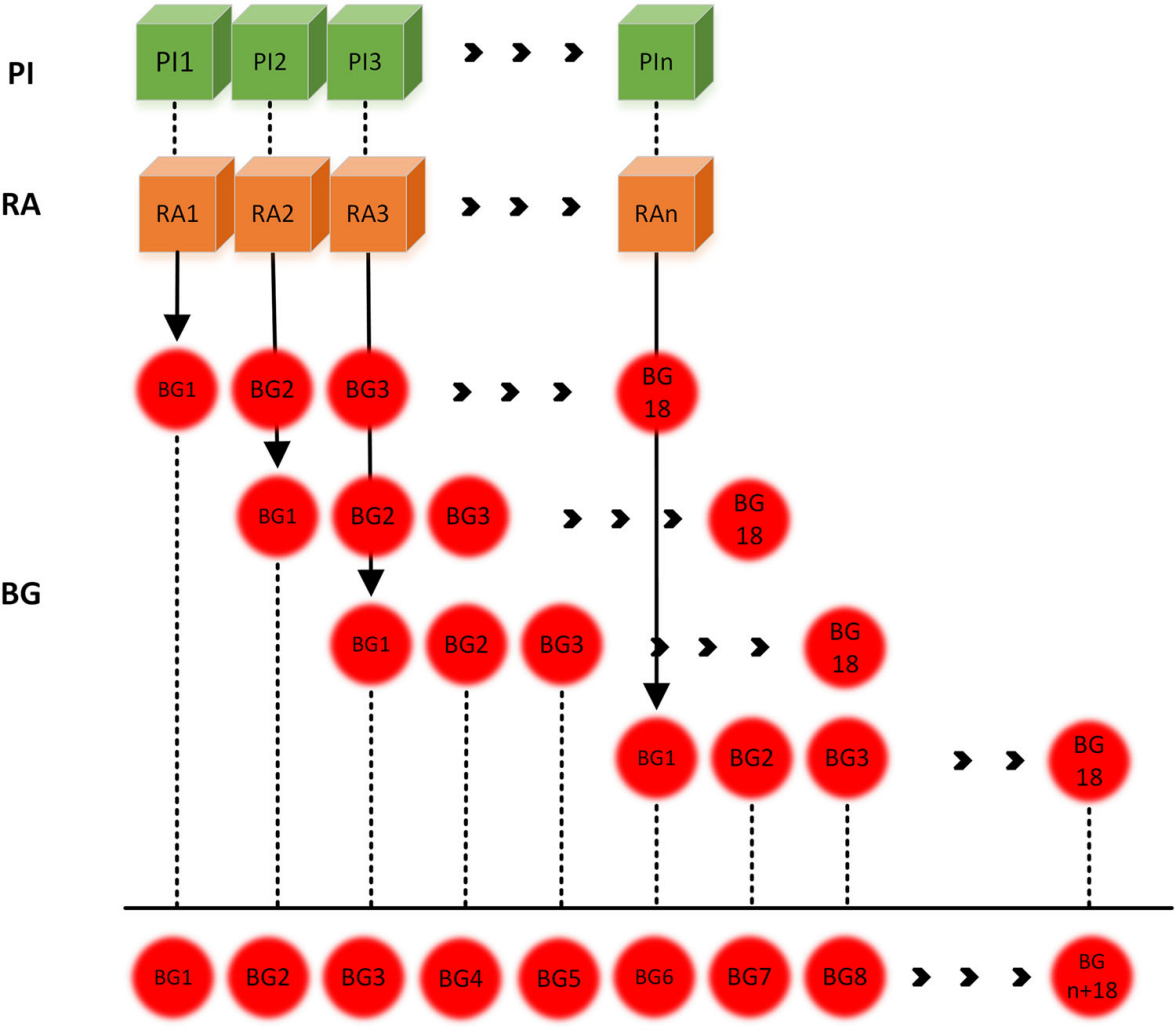
Data generation using the trained model. The phenomena of recurrence in the generated blood glucose samples: each output blood glucose stream is shifted by 1 sample for every input sample pair (PI: plasma insulin approximation, RA: carbohydrate rate of appearance, BG: blood glucose).

The trained models were then used to generate novel BG samples when provided with unseen PI and RA values from T1D patients. During generation, 1 sample of input PI and RA produced 18 samples of BG. For a series of insulin and carbohydrate values, the outputs were shifted by one sample for every input pair and then averaged to compute a recurrent output that depicted a causal relationship between the input and the output. The rationale behind this generation process came from the continuity of the latent space, which essentially means that samples that lie closer to each other in the latent space will generate outputs that are similar to each other. This phenomenon can be understood by looking at Fig. 2. With the help of this generation technique, a virtual patient was generated for every real patient, utilizing the real patient’s PI and RA information.

#### Latent space exploration

Since we know from the generation operation of a GAN that a latent space sample is transformed into the value of choice, the latent space is a very important parameter. Here, it is important to concede that latent space exploration is not presented as a unique result of this research work; however, the potential of latent space exploration is discussed for the interest of readers and as a possible future research direction in this line of research. The choice of latent space was a normal distribution in our implementation. As evident from the literature, the quality of generated data of a GAN depends on the dimensions of the latent space^62,63^. Even though there are no standards for latent space dimensions, a size of 100 or 512 is preferred in image generation tasks. For our application, it was observed that latent dimensions notably smaller than 100 produced plausible results. Moreover, the exploration of the latent space was performed during the inference phase when novel BG profiles were generated for each patient. Latent space exploration was performed using vector arithmetic. Along with generating realistic samples, varying effects of latent space exploration were observed. It was observed during this exploration that the variability of the generated BG profile could be controlled by changing the magnitude of the random samples acquired from the latent space. Larger magnitudes tended to produce outputs with a higher coefficients of variation (CVs) and vice versa. It is important to mention here that all virtual patients were generated using the same latent space configuration.

### Simulation environment

**Fig. 3:**
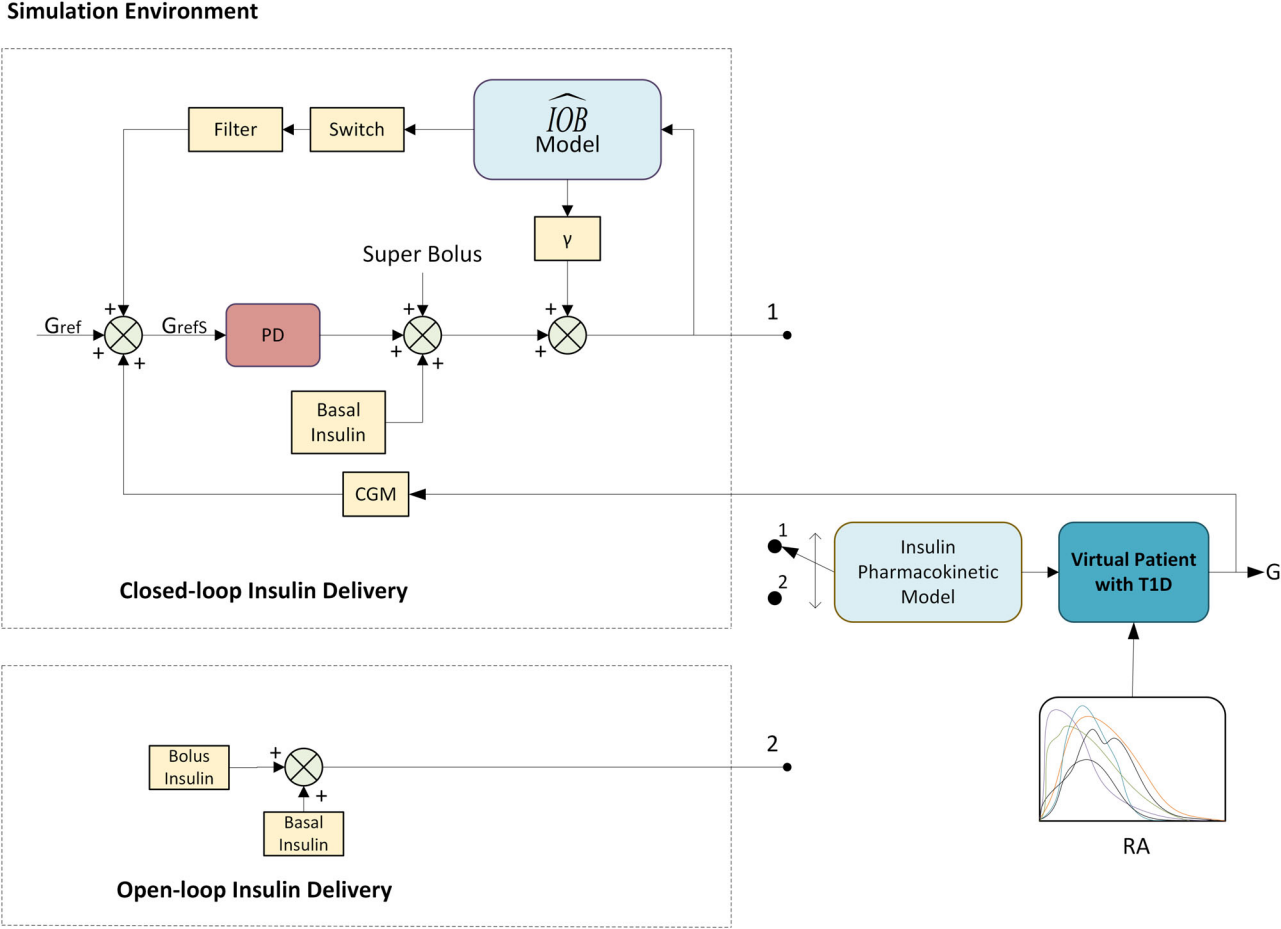
The simulation environment. Simulation environment to evaluate the closed-loop and open-loop insulin delivery systems in virtual T1D patients (IOB: insulin on board, Gref: reference blood glucose, GrefS: adjusted blood glucose, CGM: continuous glucose monitor, *γ*: insulin feedback gain parameter, RA: carbohydrate rate of appearance, PI: plasma insulin approximation).

The simulation environment shown in Fig. 3 was established for the evaluation of open-loop and closed-loop insulin delivery strategies in patients with T1D. This also served as the validation phase for the trained deep generative model. The evaluation was performed by observing the glycaemic outcomes of the generated virtual patients. These glycaemic outcomes include time-in-range results for each of the standard blood glucose ranges (<54 mg/dL, 54–69 mg/dL, 70–140 mg/dL, 70–180 mg/dL, 180–250 mg/dL, >250 mg/dL) in T1D management along with the means and variability outcomes^64^. In the proposed work, the main reason to opt for two insulin delivery therapies was to check the practicality of the proposed methodology and to further validate the approximation of the trained deep generative model in terms of causality and exactness. In the future, such therapies could be employed in the proposed simulation environment for the sole purpose of validating the therapy. The T1D patients in the cohort used in this study administered insulin to their bodies using insulin pumps and multiple daily injections under open-loop therapy. The insulin values from these patients, when provided as input to the proposed generative model, produced BG values with outcomes similar to the real patients.

Afterwards, a closed-loop insulin delivery strategy was adopted. For this strategy, the generated BG data were subjected to a state-of-the-art controller to emulate the closed-loop behaviour of a human pancreas. The controller’s control action focused on increasing the time-in-range of the generated BG by adjusting the insulin delivery to the generative model. The closed-loop insulin delivery part of the controller proposed by Beneyto et al.^65^ was utilized for this purpose. The feedback control action of the controller is composed of two loops, i.e., an insulin feedback loop, which is also referred to as the inner loop, comprising a proportional-derivative (PD) controller and an outer safety loop with insulin on board constraints and sliding mode reference conditioning. The total insulin control action is shown in Eq. (6), where *k*_*p*_ is the proportional gain, *τ*_*d*_ is the derivative time, 12C is the insulin to CHO ratio, CF is the correction factor, *G*_*ref*_ is the reference glucose value, and M is the meal carbohydrate content in grams. Here, *u*_*b*_ is the super bolus that is defined by Eq. (7). Three insulin signals constitute the inner control loop of the controller: the basal insulin profile of the patient, the super bolus, and the PD control action. Basal profiles from actual patients were used in this loop. The outer safety loop is defined to compute the conditions under which the reference glucose G*ref* needs to be changed. This is done to cease insulin infusion to keep the insulin on board (IOB) bounded, i.e., IOB ∈ [0, $\overline{IOB}$], where $\overline{IOB}$ is the maximum allowed IOB. The correction factor and carbohydrate ratio parameters from the actual patient cohort were used during patient synthesis under closed-loop therapy.

6 $${u}_{c}(t)={k}_{p}\left[{e}_{i}(t)+{\tau }_{d}\frac{dCGM}{dt}\right]+{u}_{basal}(t)+{u}_{b}$$

7 $${u}_{b}=\frac{M}{12C}+\frac{\int\nolimits_{t}^{t+60}{u}_{basal}dt}{60}M+\frac{CGM(t)-{G}_{ref}}{CF}$$

Since the generated BG data are conditioned on both insulin and carbohydrate values, the trained generative model has to have an input for both the insulin and carbohydrates to generate new BG samples. In the closed-loop therapy case, the carbohydrate data are taken from the actual cohort of patients, such as the quantity of meals in carbohydrate units consumed by a patient in real life being translated into the RA value using the meal library referenced in the methodology section. This is done to generate comparable profiles under both open-loop and closed-loop therapies using the same meal information. Moreover, the insulin input of the generative model is fed by the closed-loop controller. Because the input insulin and the generated BG depict a causal relationship, altering the amount of insulin alters the output BG values.

**Fig. 4:**
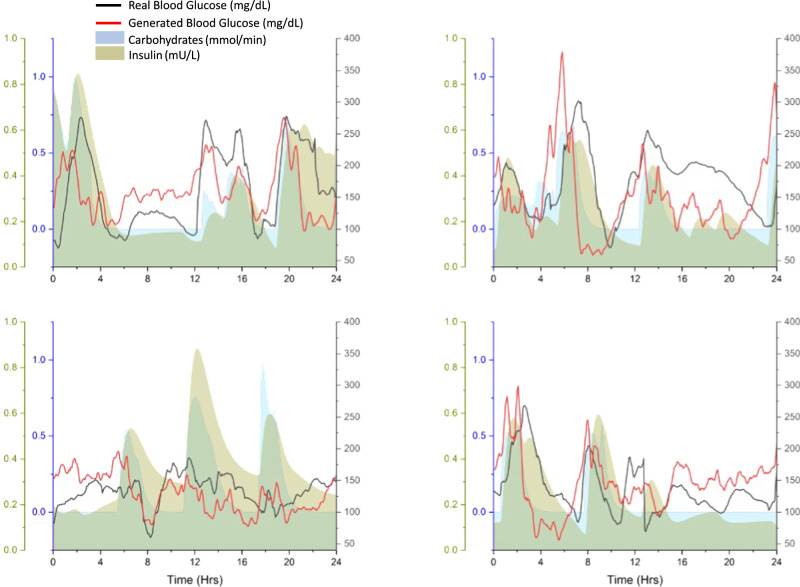
One day of generated blood glucose data vs. real blood glucose data. Twenty-four hours of generated blood glucose vs real blood glucose of patients 1, 5, 8, and 12 for the same plasma insulin approximation and carbohydrate rate of appearance inputs under open-loop therapy. The plasma insulin values are normalized between 0 and 1 mU/L whereas the carbohydrate rate of appearance values are normalized between 0 and 1 mmol/min.

To illustrate the dependence of generated BG profiles on the input PI and RA, Supplementary Fig. 4 shows a total of 2 days of BG data for four different patients. It is clear from these plots that the generated BG values take on trajectories guided by the input insulin and carbohydrate values. Similar relationships are observed in the BG data of real-life patients. These results make our proposed model unique in terms of the lifelike behaviours of the generated BG values. To reinforce this point further, the generated curves for four days of four randomly selected patients on open-loop therapy are plotted against the real curves for the corresponding days in Fig. 4. It could be observed from these curves that even though the glycaemic trends are similar in the real and simulated profiles, the values are not exactly the same. This confirms the inherently stochastic nature of the trained models, which is a desired quality of any simulation environment.

### Reporting summary

Further information on research design is available in the Nature Portfolio Reporting Summary linked to this article.

## Results

The results section is divided into three subsections. The first subsection is dedicated to the results of statistical tests performed to check the statistical similarity of the generated data to the real data. The second subsection contains results about the causal relationships between the input and output data. The third subsection presents the results of the distance test performed to measure the similarity of the probability distributions of the generated patients against the real patients.

### Statistical similarity

Data were generated for the three cohorts under open-loop therapy. However, only the carbohydrate data from the Hospital Clinic de Barcelona T1D dataset were used to generate patients under closed-loop therapy. A total of 4 weeks of BG data was generated for each patient under both open-loop and closed-loop therapies for the purpose of attaining the optimal amount of data required to report glycaemic outcomes^66^. After computing the glycaemic outcomes for each patient in both real and generated cohorts, the medians of these values along with the interquartile ranges (IQR) were computed for both cohorts. The medians of both datasets were comparable for each of the glycaemic outcomes. The Wilcoxon signed-rank test was used to evaluate statistical similarity by assuming that both the real and generated glycaemic outcomes came from the same population of patients. Based on the rejection hypothesis, all the glycaemic outcomes qualified the test by demonstrating *P* values of 0.05 or more. Tables 1, 2, and 3 present the results for the generated and real patients along with their P scores under open-loop therapy for the Hospital Clínic de Barcelona T1D dataset, the Hypomin dataset, and the Ohio dataset, respectively. The obtained statistical scores demonstrate that the glycaemic outcomes, such as BG time in standardized ranges, mean, CV, and standard deviation (STD), all show significant statistical similarity to those of the real T1D patients. These results confirm the accurate approximation of the T1D cohort by the generative model since the same insulin and carbohydrate inputs yielded similar BG outcomes.

**Table 1:** Hospital Clínic de Barcelona T1D dataset: statistical comparison of the glycaemic metrics of real patients against the generated patients: standard BG ranges in T1D management, average BG (Mean), standard deviation (STD), coefficient of variation (CV)

Glycaemic metrics	Real patients (open-loop therapy)	Generated patients (open-loop therapy)	*P*-Values
% time CGM <54	0.43 (0.13–0.90)	0.40 (0.0–2.89)	0.25
% time CGM 54–69	2.39 (1.24–3.65)	2.45 (0.43–5.67)	0.50
% time CGM 70–140	40.57 (30.07–45.68)	42.80 (30.32–54.94)	0.98
% time CGM 70–180	64.02 (55.08–68.87)	67.28 (52.85–75.70)	0.74
% time CGM 180–250	24.28 (18.79–30.08)	19.89 (11.59–29.57)	0.63
% time CGM >250	6.41 (3.45–11.43)	7.19 (1.93–13.29)	0.39
Mean CGM	157.06 (148.66–172.32)	154.20 (127.92–178.52)	0.85
STD	56.57 (50.20–64.49)	61.49 (49.07–70.48)	0.34
% CV	35.53 (32.94–39.03)	38.58 (31.81–42.60)	0.22

**Table 2:** Hypomin dataset: statistical comparison of the glycaemic metrics of real patients against the generated patients: standard BG ranges in T1D management, average BG (Mean), standard deviation (STD), coefficient of variation (CV)

Glycaemic metrics	Real patients (open-loop therapy)	Generated patients (open-loop therapy)	*P*-Values
% time CGM <54	3.61 (3.16–4.40)	2.07 (0.57–3.74)	0.131
% time CGM 54–69	4.86 (4.15–5.85)	4.60 (1.10–5.49)	0.193
% time CGM 70–140	31.75 (28.94–35.34)	29.85 (25.11–31.57)	0.275
% time CGM 70–180	49.62 (46.64–56.03)	49.34 (44.64–51.37)	0.492
% time CGM 180–250	24.70 (22.91–26.67)	25.75 (21.54–30.00)	0.375
% time CGM >250	13.76 (11.08–17.08)	11.59 (10.59–15.77)	0.922
Mean CGM	161.80 (156.05–174.28)	165.60 (154.67–180.80)	0.625
STD	76.99 (70.46–80.41)	75.79 (70.59–79.21)	1.000
% CV	45.54 (43.86–46.91)	45.84 (36.39–51.22)	0.625

**Table 3:** Ohio dataset: statistical comparison of the glycaemic metrics of real patients against the generated patients: standard BG ranges in T1D management, average BG (Mean), standard deviation (STD), coefficient of variation (CV)

Glycaemic metrics	Real patients (open-loop therapy)	Generated patients (open-loop therapy)	*P*-Values
% time CGM <54	0.31 (0.17–1.21)	0.30 (0.21–0.42)	0.519
% time CGM 54–69	2.32 (1.61–3.36)	1.83 (1.53–2.06)	0.204
% time CGM 70–140	39.49 (36.97–49.81)	40.82 (38.39–43.23)	0.850
% time CGM 70–180	65.58 (59.93–72.59)	66.67 (63.70–69.24)	0.677
% time CGM 180–250	24.05 (19.13–28.12)	22.21 (21.45–23.62)	0.301
% time CGM >250	6.89 (3.69–11.87)	7.12 (6.67–10.96)	0.791
Mean CGM	155.55 (145.52–167.18)	157.72 (154.75–167.47)	0.470
STD	57.96 (53.41–62.08)	59.08 (58.01–67.54)	0.110
% CV	36.89 (34.08–39.96)	38.25 (36.46–40.12)	0.151

During closed-loop therapy, the PD controller provided insulin as input to the generative model, while the carbohydrate data used were from real patients. The glycaemic outcomes from closed-loop therapy in terms of the medians and IQR of all the metrics for each generated patient are presented in Table 4. As per the discussion above, the control action of the controller was designed to increase the percentage time in the 70–180 mg/dL range of generated BG profiles. The glycaemic outcomes showed that the proposed model behaved in a way similar to real T1D patients by demonstrating TIR results that were closer to real scenarios. Furthermore, it has been observed in T1D patients that strong glycaemic control is often the reason for the occurrence of iatrogenic hypoglycaemia^67^. A similar phenomenon was observed in our generated patients under closed-loop therapy, and an increase in the percentage of time in level 2 hypoglycaemia was observed.

**Table 4:** Glycaemic outcomes of the generated patients under closed-loop insulin therapy: standard BG ranges in T1D management, average BG (Mean), standard deviation (STD), coefficient of variation (CV)

Glycaemic metrics	Generated patients (closed-loop therapy)
% time CGM <54	0.79 (0.07–1.88)
% time CGM 54–69	1.85 (0.57–4.23)
% time CGM 70–140	35.46 (21.47–49.84)
% time CGM 70–180	74.67 (57.03–80.70)
% time CGM 180–250	13.99 (8.30–32.37)
% time CGM >250	5.31 (2.45–9.29)
Mean CGM	155.13 (143.21–173.41)
CV	35.60 (31.92–41.01)
STD	58.11 (47.18–62.62)

### Causality analysis

In the context of a simulation environment, the model is expected to respond to the effects of certain inputs in a causal manner. The causal relationship between the inputs and outputs of a model is the basis for validating real-life phenomena using a simulation environment. There are several ways to test for causality in systems. Two of the more famous causality tests are the Granger causality test and convergent cross mapping (CCM). The Granger causality test has been used extensively by researchers for causality analysis in a variety of applications. The intuition behind this test is that if variable *X* causes variable *Y* in a system, there should be a model of such a system that improves the prediction of *Y* after the inclusion of *X*. This means that *X* should be separable from the remaining system’s variables. This is, however, a limitation of the Granger test, since in many systems with interacting variables, the information of a variable may not be separable from other variables. This poses a problem when causality is checked using the Ganger causality test in highly complex dynamical systems such as the human glucose-insulin system. CCM, on the other hand, is a causality testing methodology that identifies causalities in a system whose variables are inseparable. In addition, CCM can quantify weak to moderate causalities that other causality tests may miss. In the past, several studies have utilized CCM for causal analysis in nonlinear systems^68,69^. A recent study by Hoda et al. demonstrated the use of CCM for causality analysis in T1D^70^.

**Fig. 5:**
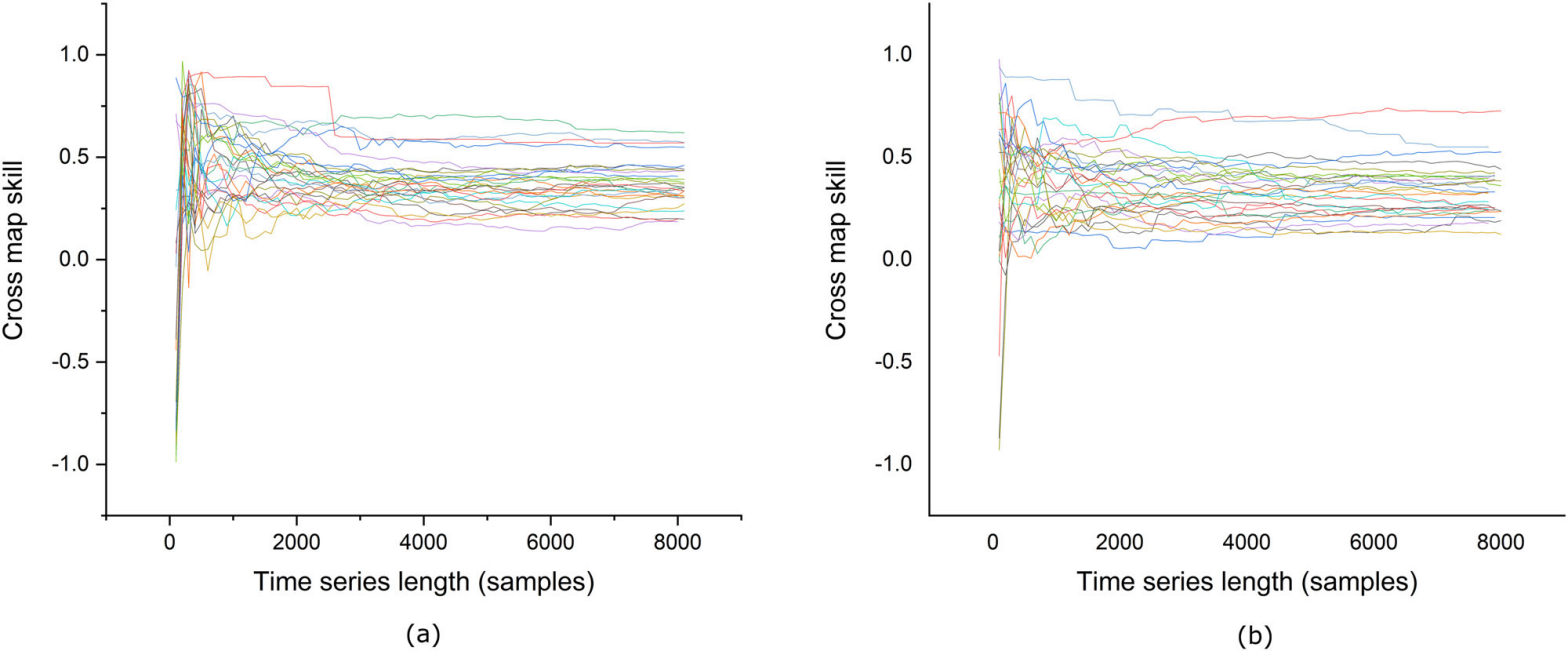
The cross-map skills for the effect of input on the generated blood glucose profiles. **a** The effect of insulin on generated blood glucose. **b** The effect of carbohydrates on generated blood glucose. The cross-map skills are shown for the 27 patients in the Hospital Clínic de Barcelona T1D dataset as a function of the time series length. A value of cross-map skill is computed after 100 samples.

**Fig. 6:**
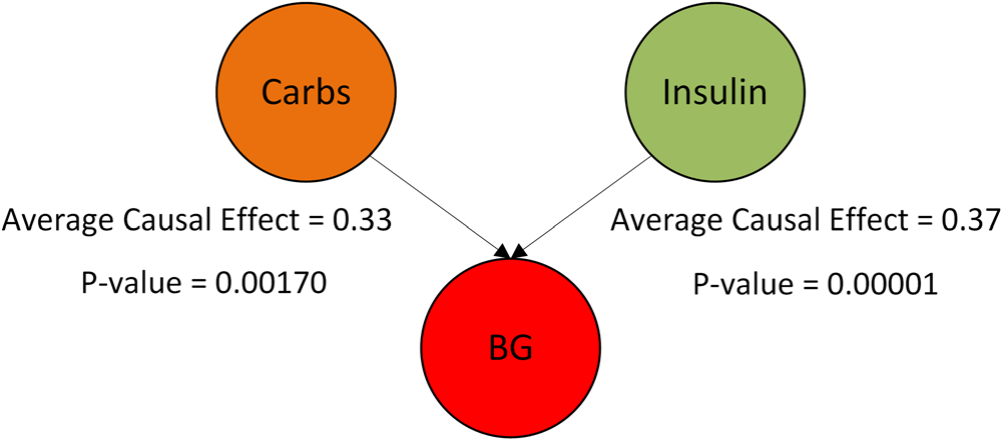
The average causal effect of carbohydrates and insulin on the generated blood glucose profiles. The average causal effect is shown for the 27 patients in the Hospital Clínic de Barcelona T1D dataset.

We used both the Granger causality test and CCM to demonstrate that the BG profiles generated using our proposed model are causally dependent on the input insulin and carbohydrate values. The causality analysis is performed for the 27 patients from the Hospital Clínic de Barcelona T1D dataset since it is the largest dataset used in this work. The results of both these tests are provided in Supplementary Tables 1 and 2. For the Granger causality test, the *P* values in Supplementary Table 1 show that both the insulin and carbohydrate values impact the generated BG values. On the other hand, as the name suggests, CCM checks for two parameters while establishing causality between two quantities, i.e., cross-mapping and convergence. Cross-mapping is measured using the correlation strength, whereas convergence is checked by observing the cross-map skill against the increasing amount of data. Even though a stronger correlation suggests stronger causation, a relationship is deemed causal only if the correlation converges as the amount of data increases. The causal strengths for individual patients are given in Supplementary Table 2, whereas the convergence is shown for all the patients in Fig. 5. It could be observed from these figures that for both insulin and carbohydrates, the cross-map skill converges as the amount of data increases. This confirms the existence of a causal relationship between the generated BG and insulin and carbohydrates. Figure 6 shows the average causal strength of insulin and carbohydrates on the generated BG values obtained using CCM and the average *P* values obtained using the Granger causality test.

### Probability distribution comparison

To further assess the similarity of the generated cohort against the real cohort, the BG time series of each individual patient of the Hospital Clínic de Barcelona T1D dataset was compared to the corresponding real patient using the Jensen-Shannon divergence (JSD) measure. JSD is a distance measure used to compute the similarity between two probability distributions, i.e., *P* and *Q*. Equation (8) shows the mathematical description of how the JSD value is computed. The resultant value of JSD falls between 0 and 1, where 0 means that the distributions are identical. This essentially means that the smaller the value of JSD is, the higher the similarity. Supplementary Table 3 gives the JSD values for all the generated patients compared to the real patients. It can be observed that the JSD value for every patient is lower, and the real and generated probability distributions are similar.

8 $${D}_{JS}=\frac{1}{2}{D}_{KL}\left(P| | \frac{P+Q}{2}\right)+\frac{1}{2}{D}_{KL}\left(Q| | \frac{P+Q}{2}\right)$$

## Discussion

The aptness of the proposed methodology for the purpose of generating glycaemic trends similar to those of real-life T1D patients has been demonstrated by the results. Approximating the human glucose-insulin system is considered the hardest part of creating a diabetes simulator. Formerly, the techniques used for these approximations were heavily based on mathematical physiological models. Apart from the advantages they offer, these models suffer from shortcomings that may lead to inaccurate approximations. Deep generative models can provide a solution for this problem. The ability of deep generative models to learn from the distribution of data obtained from a system gives them a important advantage over physiological models in that they can encompass almost all the characteristics of the system, including minor behaviours. This makes a full approximation of the system possible. Moreover, since the proposed methodology not only generates T1D patients who are similar to a cohort of real patients but can also demonstrate the behaviours of insulin, carbohydrates, and BG relationships found in real patients, the generated patients can be used in various simulation scenarios. In addition, the causality between insulin, carbohydrates, and glucose generation is shown by BG values generated in the future, which fulfils the benchmark of prediction in simulation environments. The deep generative model employed in the proposed work is a modified version of the P2P GAN architecture. Apart from redesigning the model for time-series data, several architectural changes were made to the original P2P GAN architecture. The generator architecture in the original implementation used a U-net configuration; however, since the proposed model was trained on one-dimensional numerical data, a feedforwards generator architecture was used in the proposed implementation. 1D convolutional layers were preferred over 2D convolutional layers to capture the time series properties in the data. A Wasserstein loss was employed instead of the binary cross-entropy loss along with the training recommendations of the WGAN to ensure stable training. Furthermore, the L2 distance was preferred over the L1 distance used in the original architecture because of better quality outputs.

Even though the generated BG profiles are tested for similarity in glycaemic outcomes using statistical tests, the generative model is further scrutinized using a closed-loop controller to imitate a closed-loop insulin therapy setup. This was done to further validate the causal generation of BG values by observing whether the generated BG values demonstrated the same response behaviour to closed-loop therapy as that observed in real patients. Since it was not possible to validate the closed-loop glycaemic outcomes for the real patient cohort used in this study because the patients were under open-loop insulin therapy, the only alternative method we had was to observe the glycaemic outcomes for general trends and response behaviours seen in closed-loop therapy patients. It was observed under closed-loop therapy that the proposed simulator exhibits similar glycaemic outcomes as those observed in real-life patients under closed-loop therapy^71^. This is in contrast to what is observed in conventional physiological T1D simulators where the glycaemic outcomes of patients under closed-loop are unrealistically optimistic. According to the literature, strong glycaemic control has often been associated with the occurrence of hypoglycaemia, and this behaviour was noted in the generated patients under closed-loop insulin therapy.

The proposed methodology shows promise in setting up a T1D simulation environment for the generation of novel cohorts of patients, validation of treatment methodologies, and the formation of new therapies. Considering that BG generation is caused by the input insulin and carbohydrate values, the generation of BG data with glycaemic responses of our choice may be made possible with the help of this simulator. Moreover, by exploring and quantifying the latent space configurations, the generation of cohorts with desirable characteristics may be ensured. The proposed system may also provide leverage to augment data for specific T1D patients to obtain more data in less time or at a reduced cost. This is important because the acquisition of diabetes-related data suffers from the issue of intrapatient variability since the condition of a patient’s disease varies with time. Hence, classification/prediction techniques based on data often fail to work efficiently when trained on T1D data collected over a long period.

By introducing improved closed-loop control strategies such as carbohydrate recommendations, the glycaemic outcomes of the generated BG profiles may be further improved and used in devising various other treatments. In the current implementation of the proposed simulator, insulin and carbohydrates were selected as the two conditional variables because of their high relevancy and impact in the context of the glucose-insulin model. Moreover, the availability of the data and ease of data preparation were also conclusive factors in selecting the input variables. However, the approximation of the deep generative model may be improved by conditioning the generation of BG data on more variables, such as physical activity and stress. A proficient closed-loop control system along with an accurate T1D patient generation model may make the in silico trials of different treatment methodologies as close to real scenarios as possible. Moreover, BG profiles generated by a model with a high level of approximation accuracy will present the same challenge as BG profiles from real patients for data-based techniques/models. This will ensure the development of more robust models. It will also allow designers and practitioners to be more creative and will provide confidence that the simulated scenarios will stay close to real scenarios and will not diverge to create unattainable situations. Furthermore, the evidence suggests that the proposed methodology may enable us to replicate any cohort. This allows the creation of individualized treatments. The intrinsic random nature of the generated data with the proposed model allows the generation of patients with different BG profiles and similar glycaemic characteristics. In addition, altering the latent space allows the generation of patient cohorts that may have similar glycaemic characteristics but different variability outcomes. In the future, the authors of this work are confident in producing the cohorts of choice at will to challenge the control system techniques and data-driven prediction/classification models so that they act robustly in real-life scenarios.

As evident from the theory of DNNs, the larger the amount of training data is, the better the approximation. Since they are based on DNNs, the same is observed for deep generative models. However, it is important to realize that there is no optimal amount of data for training DNNs to achieve realistic outcomes. In the proposed methodology, realistic BG generation was made possible using 1120 days of data from 27 T1D patients, 948 days of data from 10 T1D patients, and 468 days of data from 12 patients. Nonetheless, it was learned empirically that increasing the amount of training data for a particular cohort resulted in a better approximation of the dataset. This has also been proven in our prior works on the generation of BG profiles using deep generative models^15,42^. As the glycaemic behaviours of T1D patients vary greatly over the course of their lives, in a particular time phase, the generation of data to replicate a patient’s physiology will address the issue of scarce data. In the long term, the approximation of a patient’s glucose-insulin system, however, will suffer from glycaemic variability. This could pose a problem for in silico longitudinal trials. Current evidence shows that deep generative models are capable of learning the underlying distribution of any type of data and are robust to mistaken confidence errors. This suggests that the proposed method could be utilized equally well for any sort of cohort. As patients from a single cohort often exhibit similar characteristics, it is understood that training the proposed deep generative model on data from a single T1D cohort led to a good approximation of the cohort. We believe that heterogeneous training data, such as data from various different cohorts or radically different patients, may adversely affect the approximation. This essentially means that for a deep generative model-based T1D simulator to replicate the outcomes of a particular cohort of patients, it is best to train it on data from the specific cohort. However, for a more diverse simulator, the training should be performed on data from a diverse set of cohorts. This would, however, mean that the approximation of a particular cohort in the set of cohorts might not be as good.

## Conclusion

To conclude, this research study presents an AI-based T1D simulation environment based on the distribution approximation capability of deep generative models. The proposed simulator employs a sequence-to-sequence generative adversarial network for the generation of synthetic T1D patients and shows realistic results under both open-loop and closed-loop therapies. The generated data display causal relationships between the input insulin and carbohydrate values and the output blood glucose values. Moreover, the data are generated for 90 min in the future for each input, which introduces predictability in the simulation environment. In the future, the inclusion of more conditional inputs may result in improved approximation. Textual information could also be used as a conditional variable to generate data with characteristics described in the text. This may open doors for the translation of written clinical protocols into synthetic patient cohorts that fulfil the criteria defined in the protocol. The proposed technique has the potential to be leveraged for the creation of personalized digital twins, which may in turn be integrated into platforms to educate people with T1D. In addition, latent space exploration in this domain may lead to the generation of cohorts with desired characteristics. Moreover, improved control therapies could be integrated with the proposed generative model for the testing and development of new therapeutic strategies in the field of artificial pancreas development.

### Supplementary information

Supplementary Information

Description of Additional Supplementary Files

Supplmentary Data 1

Supplementary Data 2

Supplementary Data 3

Reporting Summary

## Data Availability

The Ohio T1DM dataset used in this work is available online at: http://smarthealth.cs.ohio.edu/OhioT1DM-dataset.html. The Hospital Clinic de Barcelona T1D dataset and the Hypomin dataset contain sensitive and proprietary data collected as part of clinical trials involving multiple entities. While making datasets publicly available is often beneficial for scientific progress and transparency, in the case of the Hospital Clinic de Barcelona T1D dataset and Hypomin dataset, the sensitive nature of the data, legal constraints, and ethical considerations surrounding patient privacy and collaboration agreements necessitate that they remain restricted to authorized users with appropriate permissions. The Hospital Clínic de Barcelona T1D dataset and Hypomin dataset will be made available upon reasonable request. The source data underlying Figs. 4, 5, and Supplementary Fig. 4 are provided as Supplementary Data 1, Supplementary Data 2, and Supplementary Data 3, respectively.

## Code availability

While the executable version of our software has been made freely available, we have opted to keep the underlying source code private in consideration of intellectual property rights and our business strategy. The executable version of the simulator with a sample dataset has been made available at ref. ^72^. In the event of any inquiries concerning the code, readers are encouraged to reach out to the corresponding author.
