# Supplementary material for: Generative deep learning for the development of a type 1 diabetes simulator
Source: Commun Med (Lond). 2024 Mar 16;4:51. doi: 10.1038/s43856-024-00476-0 (PMC10944502; doi:10.1038/s43856-024-00476-0)
Supplement: Supplementary file 1 — Supplementary Information [file 43856_2024_476_MOESM1_ESM.pdf]

Supplementary Information

Generative deep learning for the development of a type 1 diabetes simulator

Omer Mujahid, Ivan Contreras, Aleix Beneyto and Josep Vehi

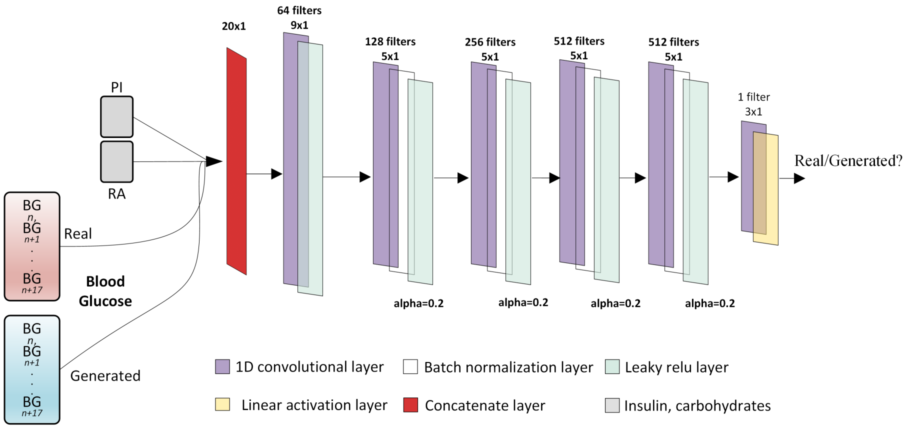

Supplementary Figure 1: Graphical depiction of the discriminator model.
Legend: The discriminator model is a binary classifier that distinguishes between real and generated blood glucose vectors (18 samples) given a particular pair of insulin and carbohydrates. (PI: plasma insulin approximation, RA: carbohydrate rate of appearance, BG: blood glucose)

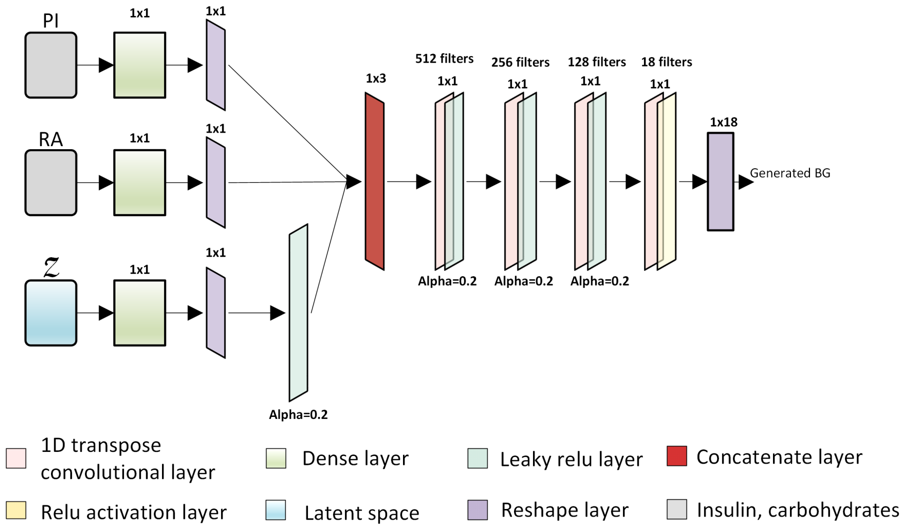

Supplementary Figure 2: Graphical depiction of the generator model.
Legend: The generator model takes the plasma insulin approximation, carbohydrate rate of appearance, and latent space values as input to generate vectors (18 samples) of blood glucose. (PI: plasma insulin approximation, RA: carbohydrate rate of appearance, Z: latent space).

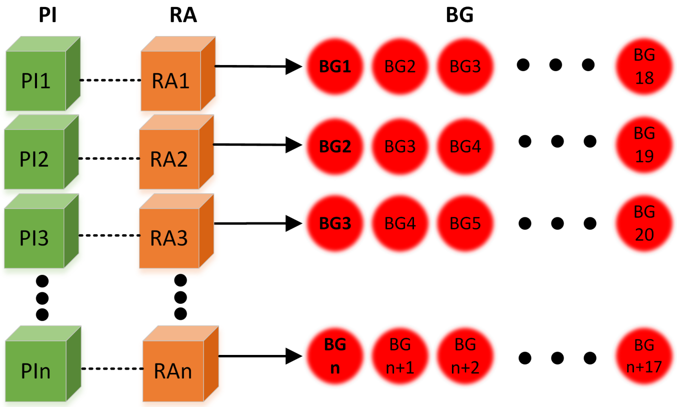

Supplementary Figure 3: The shifted plasma insulin approximation/carbohydrate rate of appearance and blood glucose pairs used to train the S2S GAN
Legend: Each input sample pair of insulin and carbohydrates corresponds to 18 output samples of blood glucose. (PI: plasma insulin approximation, RA: carbohydrate rate of appearance, BG: blood glucose).

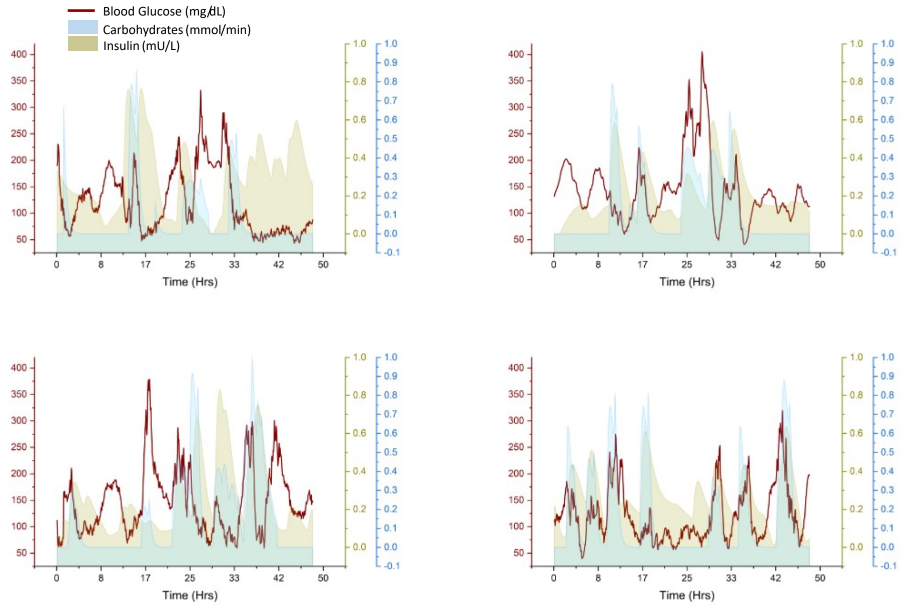

Supplementary Figure 4: Two days of generated blood glucose data of four different patient.
Legend: 48 hours of generated blood glucose data of patients 6, 15, 23, and 26 conditioned on plasma insulin approximation and carbohydrate rate of appearance under open-loop therapy. The plasma insulin approximation values are normalized between 0 and 1 mU/L whereas the carbohydrate rate of appearance values are normalized between 0 and 1 mmol/min.

Patient	Carbohydrates → BG	Insulin → BG
P1	0.00960	0.00001
P2	0.04060	0.00000
P3	0.04140	0.00000
P4	0.00170	0.00000
P5	0.00010	0.00002
P6	0.08220	0.00000
P7	0.03210	0.00000
P8	0.00000	0.00005
P9	0.01260	0.00000
P10	0.00050	0.00001
P11	0.00000	0.00000
P12	0.00060	0.00004
P13	0.00090	0.00000
P14	0.00170	0.00000
P15	0.00000	0.00000
P16	0.00010	0.00000
P17	0.01670	0.00000
P18	0.01690	0.00000
P19	0.00000	0.00000
P20	0.03680	0.00000
P21	0.00070	0.00000
P22	0.00140	0.00005
P23	0.00840	0.00000
P24	0.00000	0.00000
P25	0.00000	0.00000
P26	0.03470	0.00000
P27	0.00280	0.00000
Average	0.00170	0.00001

Supplementary Table 1: P values obtained using the Granger causality test considering that the alternative hypothesis is true.

Patient	Carbohydrates $\rightarrow$ BG	Insulin $\rightarrow$ BG
P1	0.19	0.20
P2	0.25	0.20
P3	0.20	0.55
P4	0.41	0.62
P5	0.18	0.19
P6	0.32	0.31
P7	0.25	0.24
P8	0.39	0.35
P9	0.38	0.37
P10	0.24	0.33
P11	0.55	0.57
P12	0.40	0.39
P13	0.44	0.44
P14	0.72	0.57
P15	0.33	0.41
P16	0.24	0.33
P17	0.41	0.43
P18	0.12	0.27
P19	0.28	0.31
P20	0.24	0.30
P21	0.42	0.44
P22	0.33	0.31
P23	0.34	0.32
P24	0.36	0.37
P25	0.24	0.35
P26	0.24	0.34
P27	0.53	0.46
Average	0.33	0.37

Supplementary Table 2: Causality strength values for each patient obtained using CCM.

Patient	JSD Score
P1	0.0189
P2	0.0212
P3	0.0313
P4	0.0169
P5	0.0149
P6	0.0213
P7	0.0215
P8	0.0198
P9	0.0305
P10	0.0244
P11	0.0214
P12	0.0243
P13	0.0198
P14	0.0135
P15	0.0258
P16	0.0255
P17	0.0181
P18	0.0157
P19	0.0215
P20	0.0275
P21	0.0253
P22	0.0148
P23	0.0247
P24	0.0263
P25	0.0127
P26	0.0402
P27	0.0314

Supplementary Table 3: JSD scores comparing generated patients against real patients.
