# Supplementary material for: Generative deep learning for the development of a type 1 diabetes simulator
Source: Commun Med (Lond). 2024 Mar 16;4:51. doi: 10.1038/s43856-024-00476-0 (PMC10944502; doi:10.1038/s43856-024-00476-0)
Supplement: Supplementary file 2 — Description of Additional Supplementary Files [file 43856_2024_476_MOESM2_ESM.pdf]

Description of Additional Supplementary Files

File Name: Supplementary Data 1
Description: Source data underlying Figure 4

File Name: Supplementary Data 2
Description: Source data underlying Figure 5

File Name: Supplementary Data 3
Description: Source data underlying Supplementary Figure 4
